# PSYCHOMETRIC PROPERTIES OF THE CHANGING TALK ONLINE TRAINING PROGRAM EVALUATION SCALE FOR LONG-TERM CARE STAFF

**DOI:** 10.1093/geroni/igad104.0557

**Published:** 2023-12-21

**Authors:** Frances Yang, Carissa Coleman, Clarissa Shaw, Yelena Perkhounkova, Maria Hein, Kristine Williams

**Affiliations:** University of Kansas, Kansas City, Kansas, United States; University of Kansas, Kansas City, Kansas, United States; University of Iowa, Iowa City, Iowa, United States; University of Iowa, Iowa City, Iowa, United States; University of Iowa, College of Nursing, Iowa City, Iowa, United States; University of Kansas Medical Center, Kansas City, Kansas, United States

## Abstract

The Changing Talk (CHAT) intervention educates long-term care staff about elderspeak and person-centered communication strategies. The online version of CHAT training is a national trial to reduce behavioral symptoms in long-term care residents with Alzheimer’s Disease and other dementias. The development and validation of program evaluation scales are important for ensuring valid intervention evaluation. Using item response theory to assess the satisfaction of the CHATO program by staff, we tested for unidimensionality and local independence. Confirmatory factor analysis showed that there was redundancy between two items, based on the best fitting unidimensional model (Root Mean Square Error of Approximation=0.134, Comparative Fit Index=0.999). The IRT parameters showed that the highest discriminating item was the “The information in the course was clearly presented,” which also showed the highest range of difficulty parameters for the response choices. Other items in the scale were: “This course enhanced my knowledge of the subject matter,” “The information was a good refresher for me,” “I can use this training in my job,” “The resources listed in the course were useful for my job duties,” “The video clips were useful in helping me learn course materials,” “I would recommend this course to others,” “The learning activities were useful in helping me learn course materials,” and “I was satisfied with this course overall.” Differential item functioning due to race/ethnic, language, and educational achievement were examined to test for the presence of measurement noninvariance. This program evaluation scale may be utilized in other pragmatic trials as a valid measurement tool.

